# The Impact of Adolescents’ Attachment to Peers and Parents on Aggressive and Prosocial Behavior: A Short-Term Longitudinal Study

**DOI:** 10.3389/fpsyg.2020.592144

**Published:** 2020-12-23

**Authors:** Paula Vagos, Lénia Carvalhais

**Affiliations:** ^1^Portucalense Institute for Human Development (INPP), Department of Psychology and Education, Universidade Portucalense, Porto, Portugal; ^2^Center for Research in Neuropsychology and Cognitive and Behavioral Intervention (CINEICC), Universidade de Coimbra, Coimbra, Portugal

**Keywords:** peers, parents, attachment, adolescent, prosocial behavior, aggression

## Abstract

This short-longitudinal study analyzed the cross-sectional and longitudinal pathways linking adolescent’s quality of attachment to parents and peers and their practice of aggressive and prosocial behavior; it also explored the moderation effect of gender on those pathways. A total of 375 secondary school students (203 girls and 172 boys), aged between 15 and 19 years old, completed the Inventory of Parent and Peer Attachment and the Peer Experience Questionnaire - Revised twice, within a four-month gap. Using a path analyses approach, results showed that aggression and prosocial behavior were the strongest predictors of themselves overtime. Attachment to mother had a cross-sectional effect on aggression and on prosocial behavior *via* attachment to peers, and attachment to peers predicted prosocial behavior; overall, the higher the quality of attachment, the lowest the practice of aggression and the highest the practice of prosocial behavior. These effects held stable for boys and girls, though gender-based differences were found in mean levels of attachment to peers and social behaviors. Even if other variables may be in place when understanding adolescents’ social behaviors, attachment to mother and peers also seem to play a relevant role in trying to achieve safer and more positive school climates. Suggestions on how to accomplish this are shortly discussed.

## Introduction

Though recently decreasing, the practice of aggressive acts between adolescents is still a worrisome reality (Inchley et al., 2020), as it has been found to be a stable form of behavior (Scholte et al., 2007). Such acts hold the intention of causing damage to a victim and may be in the form of overt aggression (e.g., hitting, teasing, or kicking), relational aggression, which uses relationships as weapons by manipulating between-peer relationships (e.g., excluding someone from social activities), or reputational aggression, as a way of using others to damage the victim’s social reputation within the group hierarchy (e.g., telling others to dislike someone, spreading gossips or rumors; Card et al., 2008; Heilbron and Prinstein, 2008). Boys have been found across countries to practice more overt forms of aggression (Card et al., 2008; Smith et al., 2018; Inchley et al., 2020); findings on the indirect forms of aggression (e.g., relational and reputational aggression) have not been constant and may be country/culture dependent: Card et al. (2008); Inchley et al. (2020), and Smith et al. (2018) reported no meaningful gender differences across combined samples from several countries, whereas Queirós and Vagos (2016) report higher practice of relational aggression by Portuguese adolescent boys. In turn, prosocial behaviors, which have been found to be more often practiced by girls (Queirós and Vagos, 2016), are an alternative to aggression that allow mending harmed relationship (Stubbs-Richardson et al., 2018), and consist of positive and voluntary actions that intend to help, share or comfort others, thus providing for the well-being of everyone involved (Dunfield, 2014).

Previous research has established the association between attachment and social behaviors in the adolescent years, using samples from diverse cultural backgrounds. Attachment initially referred to an affectional bond early established between infants and primary caregivers, with its characterizing features (e.g., communication, trust, and alienation) continuing to unfold and reflect in lifelong attachment-related experiences (Waters et al., 2000). Its operationalization has since evolved to consider other attachment figures (Buist et al., 2002), towards which such attachment features may apply. So, quality of attachment may be differently established with different interaction patterns, namely parents and peers, with whom adolescents spend most of their time. Parents continue to be a source of support and protection throughout adolescent years as they adapt to respond to changing demands on the part of their adolescent offspring (Moretti and Peled, 2004; Li et al., 2020), though they now share significance with peers. Still, only a minority of works considered attachment to parents and peers simultaneously, as they may serve to better understand how adolescent aggressive and prosocial behavior unfolds.

Works on attachment to parents have consistently found that its higher quality associates with lower aggressive behavior (Ooi et al., 2006; Laible, 2007; Dykas et al., 2008;, particularly indirect aggression, de Vries et al., 2016) externalizing behavior (Allen et al., 2007), and bullying (Charalampous et al., 2018; D’Urso and Pace, 2019). About attachment to mother/father, previous evidence is inconsistent, with works pointing to the relevance of mother (e.g., DeMulder et al., 2000, though using only mothers) or father (Gallarin and Alonso-Arbiol, 2012). Attachment to peers have also been found to predict diminished practice of bullying (Burton et al., 2013; D’Urso and Pace, 2019; Schoeps et al., 2020) and increased prosocial indicators (Allen et al., 2002; Carlo et al., 2011; Schoeps et al., 2020). When attachment to mother, father and peers is investigated simultaneously in relation to adolescents’ social behaviors, more complex and inconsistent findings appear. Laible (2007) found only indirect effects linking attachment to parents and aggression, and linking attachment to parents and peers with prosocial behavior. Tambeli et al. (2012) and Oldfield et al. (2016) found that only attachment to parents (and not to peers), predicted aggression and conduct problems in adolescence, respectively. In turn, Murphy et al. (2017), Pan et al. (2017), and Malonda et al. (2019) posed that both attachment to parents and to peers predicted aggression and externalizing problems; Malonda et al. (2019) refer to the relevance of attachment to father, whereas Pan et al. (2017) report the prominence of attachment to father. About prosocial behavior, Oldfield et al. (2016) found that attachment to peers (but not parents) predicted prosocial behavior, whereas Malonda et al. (2019) propose that attachment with mother and peers associated with that behavior, and still Pan et al. (2017) found that it was not directly predicted by any form of attachment. In general, these findings seem to point to attachment to parents and peers serving different functions with regards to adolescent behavior: parents have a stronger role in relation to aggression whereas peers have a sturdier role in relation to prosocial behavior.

None of these works differentiated the forms of aggression, whose consequents have been disclosed (e.g., Prinstein et al., 2001; Card et al., 2008), but not its antecedents. Previous works using a longitudinal design, which would seem preferable to study the complex ties between attachment and adolescent behavior, further failed to differentiate attachment figures (e.g., Allen et al., 2002, 2007; Charalampous et al., 2018; Malonda et al., 2019). So, the current study used a short longitudinal design to examine the simultaneous impact of attachment to mother, father, and peers on adolescents’ practice of aggressive and prosocial behaviors. We expect that higher quality of attachment will impact in less aggressive and more prosocial behavior, either within the same timeframe (i.e., attachment and practiced behavior at time 1 or at time 2, alike, for example, Murphy et al., 2017) or over a longitudinal four-month timeframe (i.e., attachment at time 1 and practiced behavior at time 2, alike, for example, Malonda et al., 2019). We expected attachment to mother and father to be particularly associated with aggression, whereas attachment to peers might more strongly associate with prosocial behavior (alike, for example, Oldfield et al., 2016). We also considered that attachment and social behaviors should predict themselves over time, in line with previous findings (e.g., Allen et al., 2002)^1^. Finally, we explored if gender had a moderating role on the pathways linking attachment with social behaviors; previous findings on the subject considering boys and girls separately indicate such links to be stronger for girls (Nikiforou et al., 2013; You and Kim, 2016).

## Method^2^

### Participants

Participants were 375 middle and late adolescents (see Supplementary Material), aged 15–19 years old (*M* = 16.62, SD = 1.03), of which 45.9% (*n* = 172) were boys and 54.1% (*n* = 203) were girls; boys and girls had similar mean ages [*t*_(373)_ = −0.56, *p* = 0.58]. Concerning school year, 31.7% (*n* = 119) attended the 10th grade, 38.7% (*n* = 145) attended the 11th grade, and 29.6% (*n* = 111) attended the 12th grade. Most of these students had never been retained in the same school year before (*n* = 290, 77.3%), whereas 22.7% of them (*n* = 85) had been retained 1–3 times before. Boys and girls were similarly distributed by school year [χ*^2^*_(2)_ = 2.57, *p* = 0.28], though boys were more likely to have been retained than girls [χ*^2^*_(1)_ = 10.38, *p* = 0.001]. As for socioeconomic status (SES)^3^, the majority of these students descended from a medium SES (*n* = 257, 68.5%) and a minority came from a high SES (*n* = 2, 0.8%), with the remaining belonging to a low SES (*n* = 114, 30.4%). Boys and girls were distributed similarly regarding their families’ SES [χ*^2^*_(2)_ = 0.55, *p* = 0.76]. These participants were assessed two times within a four-month interval (see section “Procedures”).

### Instruments

#### Inventory of Parent and Peer Attachment (IPPA)

The IPPA intends to measure the quality of attachment to mother, father, and peers, as reflected in high levels of mutual trust and quality of communication, and low levels of anger and alienation. It uses three scales, one for each attachment figure, which have proven to be independent and internally consistent factors (i.e., α between 0.87 and 0.92), to associate positively with quality of familiar environment and positive self-concept as a family member, and to associate negatively with loneliness and hopelessness in adolescents (Armsden and Greenberg, 1987). The Portuguese version of the IPPA (Neves et al., 1999) held a three-factor exploratory solution (i.e., attachment to mother, to father, and to peers) with very good internal consistency values (i.e., ranging from 0.92 to 0.95), though some items were excluded, all due to low loading and communality values. So, attachment to mother and father measures are composed by 21 items each and attachment to peers includes 19 items, all answered using the same five-point Likert type scale (i.e., ranging from 1 – almost never or never true to 5 – almost always or always true). Using the current sample, the three-factor measurement model was confirmed (Table 1) and found to be invariant by gender for time 1 (metric invariance: Δ*CFI* = −0.005, Δ*RMSEA* = 0.000, and Δ*SRMR* = 0.009; scalar invariance: Δ*CFI* = −0.007, Δ*RMSEA* = 0.001, and Δ*SRMR* = 0.003) and time 2 (metric invariance: Δ*CFI* = −0.001, Δ*RMSEA* = 0.000, and Δ*SRMR* = 0.005; scalar invariance: Δ*CFI* = −0.01, Δ*RMSEA* = 0.001, and Δ*SRMR* = 0.002). Internal consistency values were excellent for all measures at both time points (α > 0.92; see Supplementary Table A).

**TABLE 1:** Fit indicators for the measurement model and for the path analyses.

	χ^2^	df	RMSEA	90% CI for RMSEA	CFI	SRMR
**Measurement models**						
**Time 1**						
Inventory of Parent and Peer Attachment^*a*^	4,157.66	1,764	0.060	0.058; 0.063	0.81	0.060
Peer Experience Questionnaire – bully version	104.32	71	0.035	0.019; 0.049	0.97	0.043
**Time 2**						
Inventory of Parent and Peer Attachment^*b*^	4,141.52	1,756	0.060	0.058; 0.063	0.84	0.060
Peer Experience Questionnaire – bully version	143.34	71	0.052	0.040; 0.064	0.94	0.046
**Structural equation modeling**						
Baseline model	297.01	21	0.194	0.175; 0.210	0.83	0.102
Generated model	45.86	34	0.030	0.000; 0.051	0.99	0.062
Boys	52.99	34	0.057	0.023; 0.086	0.96	0.073
Girls	51.78	34	0.051	0.018; 0.077	0.95	0.059
Unrestrictive model	116.53	60	0.067	0.048; 0.085	0.94	0.066
All pathways constrain equal model	138.16	74	0.064	0.047; 0.080	0.93	0.107
All means constraint equal model	243.02	85	0.094	0.080; 0.108	0.82	0.127

*All chi-square values were significant at *p* < 0.01.*

*^*a*^Acceptable fit indicators were obtained after allowing for two residual covariances; covariances were only allowed between items belonging to the same attachment measure, with 1 being within items measuring attachment to father and one being within items measuring attachment to mother.*

*^*b*^Acceptable fit indicators were obtained after allowing for ten residual covariances; covariances were only allowed between items belonging to the same attachment measure, with 4 being within items measuring attachment to father and 6 being within items measuring attachment to mother.*

#### Peer Experience Questionnaire – Revised (RPEQ)

The RPEQ is a self-report instrument that evaluates the adolescents’ experience of aggression, namely its practice (i.e., bully version – 14 items) and receiving; given the goals of the current work, only the bully version was used. It refers to how often adolescents engaged in an aggressive (overt, relational, and reputational) or prosocial behavior toward others in the past year, using a 5-point scale ranging from 1 (never) to 5 (a few times a week). The four-factor measurement model assumed for this measure has been replicated *via* exploratory (De Los Reyes and Prinstein, 2004) and confirmatory factor analyses, and found to be invariant across gender and schooling (i.e., participants attending middle or high school; Queirós and Vagos, 2016). Acceptable internal consistency values have been found for all bully measures, ranging from 0.68 to 0.91 (De Los Reyes and Prinstein, 2004; Queirós and Vagos, 2016). Evidence was also found in favor of the construct validity of these measures (Queirós and Vagos, 2016). Using the current sample, the four-factor measurement model was confirmed (Table 1) and found to be invariant by gender at time 1 (metric invariance, after allowing the loading of item 13 to vary between boys and girls: Δ*CRI* = −0.006, Δ*RMSEA* = 0.000, and Δ*SRMR* = 0.01; scalar invariance: Δ*CFI* = −0.01, Δ*RMSEA* = 0.002, and Δ*SRMR* = 0.003) and time 2 (metric invariance: Δ*CFI* = 0.002, Δ*RMSEA* = −0.003, and Δ*SRMR* = 0.013; scalar invariance: Δ*CFI* = −0.004, Δ*RMSEA* = −0.001, and Δ*SRMR* = 0.002). All measures achieved at least good internal consistency levels (α > 0.60; see Supplementary Table A).

### Procedures

#### Sampling Procedures

Authorization for this work was firstly obtained by the national entity responsible for the ethics of studies conducted in school settings (entry no. 0296300008), then by the executive boards of three schools from the center region of Portugal, and then from parents/legal guardians of participating students. Finally, the assent of students themselves was asked within classroom, during time made available by the teacher, upon which students were presented with the goals of the current work, its procedures, and the confidentiality and anonymity of the data they would provide. Assenting students then filled in the Portuguese versions of the self-report questionnaires described above. The first data collection time was carried out at the end of the first trimester of the school year (i.e., Time 1) and the second data collection time occurred roughly **4** months later (i.e., Time 2).

#### Statistical Analysis

Data analyses pertaining to the measurement models of each instrument (see section “Instruments”) and to the predictive path analyses were carried out using Mplus V7.4 (Múthen and Múthen, 1998–2012). A baseline model (see Supplementary Figure A) was tested and further modified to achieve both a more parsimonious and more statistically acceptable model; modifications to the model were imputed solely if they did not disrupt the direction of the predictive pathways as stated in our hypotheses. Because same timeframe and longitudinal hypotheses were tested simultaneously, the baseline model considered a direct effect of attachment on time 1 to attachment on time 2 and to social behaviors on times 1 and 2; attachment at time 1 could have an indirect effect at social behaviors at time 2 *via* attachment at time 2 or social behaviors at time 1. The model was considered to be statistically acceptable if Comparative Fit Index (CFI) value was higher than 0.92, combined with either a Standardized Root Mean Residual (SRMR) value lower than 0.08, or a Root Mean Square Error of Approximation (RMSEA) value lower than 0.06 (Hair et al., 2014).

Gender-based invariance was then investigated on the generated model. Three levels of invariance were considered: (1) configural invariance, meaning that the model was an adequate fit for boys and girls, when considered separately, (2) equality of pathways, and then (3) equality of means. For invariance to be established each equality constraints should not significantly worsen the fit of the model *via* a qui-square difference test approach.

## Results

The Full-information Maximum Likelihood Robust estimator was used, to account for deviations to the normal distribution (i.e., all Mardia’s test statistic significant at *p* < 0.001 for all measures at times 1 and 2) and for the presence of missing values^4^, which represented 2% of the total item pool and were missing completely at random [X ^2^_(474)_ = 233.72, *p* = 0.47]. Preliminary data analyses also indicate that attachment to father and peers, aggression, and prosocial behavior were strongly correlated and stable over time. Attachment to mother significantly decreased over time, though being highly correlated within the two data collection moments (see Supplementary Material).

### Path Analyses

**FIGURE 1:**
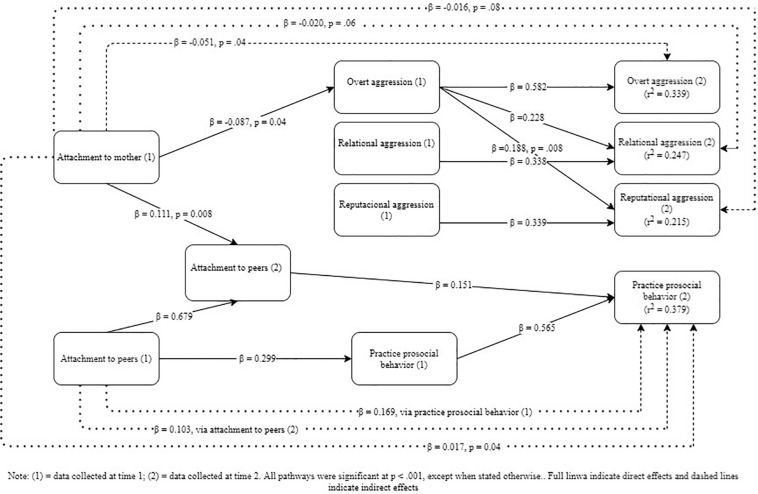
(1) = data collected at time 1; (2) = data, collected at time 2. All pathways were significant at *p* < 0.001, except when stated otherwise. Full lines indicate direct effects and dashed lines indicate indirect effects.

The baseline model was not a good fit for the data. Following a model generation approach, two steps were sequentially taken in trying to achieve a model that was both theoretically meaningful and statistically significant, namely deletion of all non-significant pathways and inclusion of correlational pathways that seemed theoretically justified^5^. The resulting model was a good fit for the data (Table 1) and is depicted in Figure 1.

In specific, attachment at time 1 had only indirect effects on aggression and prosocial behavior at time 2. Attachment to mother was particularly relevant to the diminished practice of aggression, whereas attachment to peers was especially important to the increased practice of prosocial behavior; attachment to father had no direct or indirect effect on practicing aggressive or prosocial behaviors. Alternatively, practice of overt aggression at time 1 predicted the enactment of all forms of aggression at time 2, in addition to all specific forms of behavior predicting themselves over time.

### Gender-Based Invariance

The generated model was a good fit for the data of boys and girls taken separately, thus indicating configural invariance. Full pathways invariance was also found [Δχ^2^_(21)_ = 31.29, *p* > 0.05] but no evidence was found for invariance at the means level [Δχ^2^_(11)_ = 104.87, *p* < 0.001]. So, between-gender comparisons based on non-parametric tests were carried out and further show that boys had significant higher mean values than girls for practicing all forms of aggressive behavior at both times. Girls, in turn, scored significantly higher than boys on attachment to peers at both times and on practicing prosocial behavior at time 2 (for a detailed account on gender-based invariance, see Supplementary Material).

## Discussion

The current work followed previous ones (e.g., Oldfield et al., 2016; Schoeps et al., 2020), but innovated by using a longitudinal design to explore the simultaneous pathways linking attachment to mother, father and peers to diverse practiced forms of aggression and to prosocial behavior. Current findings highlight that each behavior was the best predictor of itself (alike Allen et al., 2002) and that the frequency with which it is practiced is stable over a four-month time frame (Scholte et al., 2007). In fact, we found no direct impact of parents or peers’ attachment on interpersonal behaviors from one time point to another, which may precisely have to do with each behavior accounting for the larger amount of its variance over time. Alternatively, overt aggression predicted itself and other forms of aggression over time, indicating that it may transform as adolescents realize which aggressive acts are susceptible to punishment by the school (and family) system, thus justifying that the practice of overt forms of aggression decline with age (Inchley et al., 2020). As physical forms of aggression become increasingly punished, adolescents may turn to relational or reputational aggression and practice it more frequently, especially toward peers with whom they spend most of their time, in detriment of time spent with parents (Moretti and Peled, 2004).

Though effects of attachment on social behaviors were only indirect or cross-sectional, it is worth noticing that attachment to mother and peers impacted differently on diverse social behaviors. Quality of attachment to mother predicted lower practice of overt aggression, which resembles previous findings relating attachment to mother and aggression (DeMulder et al., 2000), externalizing problems (Pan et al., 2017) or delinquency (Allen et al., 2002). About attachment to peers, it impacted on increased prosocial behaviors in particular, which again concurs with previous findings (Laible, 2007; Carlo et al., 2011; Oldfield et al., 2016; Malonda et al., 2019; Schoeps et al., 2020). Attachment to mother also had an indirect effect on prosocial behavior *via* attachment with peers; so, quality of attachment to parents may be an asset for overall adolescent development, in as much as previous experiences with parents and/or caregivers, namely values that were acquired and internalized, will still likely emerge in peers’ relationships (Moretti and Peled, 2004). Attachment to father was not a significant predictor of practiced social behavior. Its relevance may become absent when mothers are simultaneously considered [unlike, for example Malonda et al. (2019), who considered a single parents measure] and/or when quality of attachment is analyzed, regardless of parental practices (unlike Gallarin and Alonso-Arbiol, 2012, who had those practices as independent variables and found prominence for attachment to fathers). Instead, current findings on attachment to fathers is in line with adolescents reporting that they feel more comfortable communicating with their mothers than with their fathers (Inchley et al., 2020).

The model linking attachment to aggression and prosocial behavior was found to apply similarly to boys and girls. Previous findings had pointed to diverse gender-based pathways between attachment to parents and aggression (Nikiforou et al., 2013), but similar pathways linking attachment to peers and prosocial behaviors (Schoeps et al., 2020); only the latter work considered the same multi-group analyzes methodology as we did. So, though individual gender-based models might have appeared if considering boys and girls separately, we expect a non-gender-specific model to prove more useful and informative, given that explanatory (e.g., Dodge and Rabiner, 2004) and intervention models (e.g., Boxer and Dubow, 2002) on aggression do not differentiate by gender. Mean level gender differences further concur to our instruments having evaluated their intended constructs, in as much as they align with previous literature: girls scored higher on peer attachment (Gullone and Robinson, 2005), practiced more prosocial behaviors (Stubbs-Richardson et al., 2018), and practiced less overt aggressive behavior than boys (Card et al., 2008; Inchley et al., 2020); girls also practiced less relational and reputational aggression, which may be a cultural specific finding that replicates previous ones with similar samples (Queirós and Vagos, 2016). These mean level differences may be pointing to a social profile, where one (particularly girls) is better attached (principally to peers) and practices less aggressive and more prosocial behavior. Adolescents who establish peer relationships based on prosocial behavior may have little room for quarrelsome ones, and be more prosocial in responding to bullying (Dykas et al., 2008; Stubbs-Richardson et al., 2018).

### Implications for Applied Settings

It seems relevant to promote enhanced quality of attachment to mother and peers, given that these figures had an impact on either diminished aggression or increased prosocial behavior. Attachment-based family therapy (Diamond et al., 2002) may be an option; though it has been applied especially with young children, its adaptation to adolescence seems justified. Also, school-based holistic intervention programs, that simultaneously target aggressors, victims, and bystander peers (e.g., Ikeda et al., 2004), may be a relevant way of promoting the quality of peer attachment and (consequentially) of positive interpersonal cycles in which all agents of the interaction are invested. Such positive cycles should be particularly based on diminishing overt aggression, which seems to evolve to other forms of aggression over time, and on promoting alternative behaviors to aggression, namely prosocial and assertive ones. Though our findings point to a time-limited effect of attachment on aggression and prosocial behavior, we might hope that, if these cycles are established within a school community, they may become self-sustained. In fact, peer relationships may turn out to be optimal learning experiences as to which behaviors will be socially accepted/rewarded (i.e., prosocial behaviors) versus non-accepted/punished (i.e., practiced aggressive behaviors).

### Limitations

This study relied only on self-report measures, which are susceptible to reporting bias, even when presenting adequate reliability and internal validity, which was the case for measures in our work, though borderline for relational aggression at time 1. Future studies could consider other methods of data collection, such as peer-, parents- and teacher-reports, interviews, or observational methods. It might also be important to explore other variables as they may relate to the pathways we intended to explore. For example, previous works refer to the relevance of emotional competence (Laible, 2007; You and Kim, 2016), empathy (Carlo et al., 2011; Schoeps et al., 2020) or parental practices (Gallarin and Alonso-Arbiol, 2012), but none in relation to the diverse forms of aggression. Moreover, considering other types of social behaviors, namely internalizing ones (e.g., safety-seeking behaviors), may better untangle the impact of specific attachment figures, as previous works refer to mother and father impacting differently on internalizing and externalizing problems (Galambos et al., 2003; Liu, 2008; Tambeli et al., 2012). The role of teachers as attachment figures should also be explored, as it may particularly impact social behaviors that occur mainly in between-peer interactions in school settings, as were those currently considered. Finally, considering cyber aggression, which is becoming more frequent (Inchley et al., 2020), seems warranted; previous findings have pointed to similar links between attachment and aggression or cyber-aggression (Burton et al., 2013; Charalampous et al., 2018), though the forms of aggression have not been considered.

### Conclusion

Aggression and prosocial behaviors seem to be the best predictors of themselves over a four-month timeframe; in turn, attachment to mother and peers seem to, at each time point, impact differently on those social behaviors. Specifically, attachment to mother impacted on practiced aggression whereas attachment to peers had an impact on practiced prosocial behavior. So, trying to promote higher quality of attachment to mother and peers may have a direct and same-time effect on the aggressive and prosocial acts between adolescents, which may come to be sustained over time *via* naturally occurring positive interpersonal cycles, which contribute to an overall positive and adjusted adolescent psychosocial functioning (Laible et al., 2000; Oldfield et al., 2016; Li et al., 2020).

## Data Availability Statement

The raw data supporting the conclusions of this article will be made available by the authors, without undue reservation.

## Ethics Statement

The studies involving human participants were reviewed and approved by General Directorate for Education (entry no. 0296300008). Written informed consent to participate in this study was provided by the participants’ legal guardian/next of kin.

## Author Contributions

PV was responsible for the design of the study, for describing the methods and results sections of the manuscript. LC was responsible for the introduction and discussion section of the manuscript. Both authors contributed with validating each other’s responsibilities and to the writing of the manuscript in its current presentation.

## Conflict of Interest

The authors declare that the research was conducted in the absence of any commercial or financial relationships that could be construed as a potential conflict of interest.

## Footnotes

1 For a detailed account of these hypotheses, please see Supplementary Material.

2 This project is public at https://osf.io/nkpr4/?view_only=ce86c44593484a548951ef8f59221baa

3 Socioeconomic status was measured based on parents’ profession, considering the Portuguese profession classification (Instituto Nacional de Estatística, 2011). Examples of professions in the high socioeconomic status groups are judges, higher education professors, or M.D.s; the medium socioeconomic status group includes nurses, psychologists, or school teachers; the low socioeconomic group comprises of included farmers, cleaning staff, or undifferentiated workers. One participant did not provide information on his parents’ profession, so his SES could not be inferred.

4 For a detailed account on missing values please see Supplementary Material.

5 A detailed account on excluded pathways and added correlations may be requested from the first author.
