# Supplementary material for: The Impact of Adolescents’ Attachment to Peers and Parents on Aggressive and Prosocial Behavior: A Short-Term Longitudinal Study
Source: Front Psychol. 2020 Dec 23;11:592144. doi: 10.3389/fpsyg.2020.592144 (PMC7786050; doi:10.3389/fpsyg.2020.592144)
Supplement: Supplementary file 1 [file Table_2.DOCX]

Supplementary Material

# Specific hypotheses for baseline model

# Within our goal of examining the simultaneous impact of attachment to mother, father, and peers on adolescents’ practice of aggression and prosocial behaviors, be it either within the same timeframe or over a longitudinal four-month timeframe, we posed specific hypotheses, which are listed below.

Same timeframe hypotheses

- Higher quality of attachment at time 1 will have a direct effect on less practice of aggressive behavior and more practiced of prosocial behavior at time 1.
- Higher quality of attachment at time 2 will have a direct effect on less practice of aggressive behavior and more practiced of prosocial behavior at time 2.

Longitudinal hypotheses:

- Higher practice of aggressive or prosocial behavior at time 1 will have a direct effect on its higher practice at time 2.
- Higher quality of attachment at time 1 will have a direct effect on its higher quality at time 2.
- Higher quality of attachment at time 1 will have a direct effect on less practice of aggressive behavior and more practiced of prosocial behavior at time 2.

# Sametime and longitudinal hypotheses were tested simultaneously using a path analyses approach described in the statistical analysis section of the manuscript and depicted in Figure A of this Supplementary Material. As such, several indirect effects were investigated:

- Attachment at time 2 will mediate the impact of attachment at time 1 on practice of aggressive and prosocial behavior at time 2.
- Practice of aggressive and prosocial behavior at time 1 will mediate the impact of attachment at time 1 on practice of aggressive and prosocial behavior at time 2.

Supplementary Figure A presents a simplified version of the baseline model that we proposed to test and that considered all the hypotheses stated above.

Supplementary Figure A


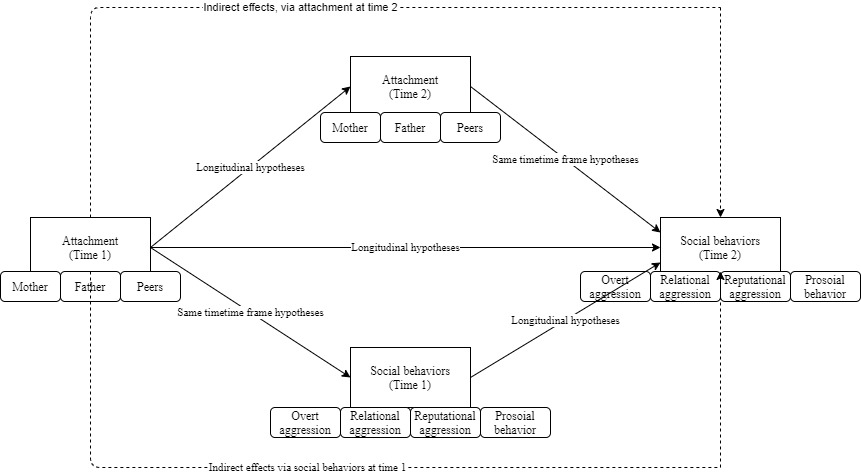


# Participants

The World Health Organization defines middle adolescents as being aged between 14 and 16 years old and late adolescents as being aged between 17 to 19 years old. This age range seems appropriate to explore the main goal of the current work (i.e., uncover the simultaneous impact of attachment to mother, father,and to peers on adolescents’ practice of aggressive and prosocial behavior) because previous evidence seems to point that only then are parents and peers considered equally as attachment figures: peers start to be considered as providers of proximity and safeness only in the later years of early adolescence (Nickerson & Nagle, 2005), to be named on equal footing as parents by late adolescents (Freeman & Brown, 2001).

References

World Health Organization (2020). Age – not the whole story. Retrieved at September 22nd 2020 at <https://apps.who.int/adolescent/second-decade/section2/page2/age-not-the-whole-story.html>

Freeman, H. & Brown, B.B. (2001). Primary attachment to parents and peers during adolescence: Differences by attachment style. Journal of Youth and Adolescence, 30(6), 653-674. <https://doi.org/10.1023/A:1012200511045>

Nickerson, A.B. & Nagle, R.J. (2005). Parent and peer attachment in late childhood and early adolescence. The Journal of Early Adolescence, 25(2), 223-249. <https://doi.org/10.1177/0272431604274174>

# Missing Values Analyses

Our initial sample consisted of 423 participants of which 135 (31.91%) presented with missing values, particularly for our independent variables (i.e., attachment to mother, father and peers at time 1); 48 participants presented with significant missing data (i.e., more then 5%) for two of those measures and so were dropped from the study. The remaining 375 participants represented our final sample, of which 87 (23.2%) presented with missing values that accounted for 2% of the total response pool and were missing completely at random (*Χ^2^*_(474)_ = 233.72, *p* = .47). Because the percentage of missing values was minimal (i.e., less than 5% for the total item pool) and randomness of missing values was established, missing values were handled via the Full-Information Maximum Likelihood estimator (Tabachnick & Fidell, 2013) in Mplus v7.4.

References

Tabachnick, B.G. & Fidell, L.S. (2013). *Using multivariate statistics* (6^th^ ed.). Pearson.

# Preliminary Results

Results indicate no significant mean differences for attachment to father or peers; attachment to mother decreased significantly from time 1 to time 2 of data collection (Supplementary table A). No significant mean differences over time were found for any form of aggressive behavior or for prosocial behavior. Moreover, each measure correlated significantly with itself over the two data collection time points.

| Supplementary table A: Internal consistency, descriptive, and correlation values for/between times 1 and 2 | | | | | | | | | |
| --- | --- | --- | --- | --- | --- | --- | --- | --- | --- |
|  | | Time 1 | | | Time 2 | | | *z* | *r_s_* between time 1 and 2 |
|  | | α | M | SD | α | M | SD |  |  |
| Inventory of Parent and Peer Attachment | |  |  |  |  |  |  |  |  |
|  | Mother | .93 | 3.86 | 0.69 | .94 | 3.81 | 0.69 | -2.33^*^ | 0.80^**^ |
|  | Father | .95 | 3.59 | 0.88 | .96 | 3.56 | 0.89 | -1.19 | 0.81^**^ |
|  | Peers | .92 | 3.84 | 0.62 | .94 | 3.81 | 0.68 | -0.21 | 0.72^**^ |
| Peer Experience Questionnaire Revised– bully version | |  |  |  |  |  |  |  |  |
|  | Overt aggression | .71 | 1.39 | 0.59 | .76 | 1.38 | 0.58 | -0.70 | 0.61^**^ |
|  | Relational aggression | .60 | 1.45 | 0.57 | .68 | 1.43 | 0.54 | -0.72 | 0.52^**^ |
|  | Reputational aggression | .79 | 1.23 | 0.51 | .83 | 1.23 | 0.57 | -0.33 | 0.38^**^ |
|  | Practice of prosocial behavior | .83 | 3.10 | 0.8 | .82 | 3.09 | 0.79 | -0.61 | 0.58^**^ |
| ^**^ p < .001, ^*^ p < .05  Note: z values refer to a mean comparison for repeated measures across times 1 and 2 | | | | | | | | | |

# Gender based-invariance analyses

Evidence was found for gender-based invariance at the pathway levels. Supplementary figures B and C show the generated model when all pathways are constraint to be equal across gender, for boys and girls respectively.

Supplementary Figure B: Generated model applied to boys


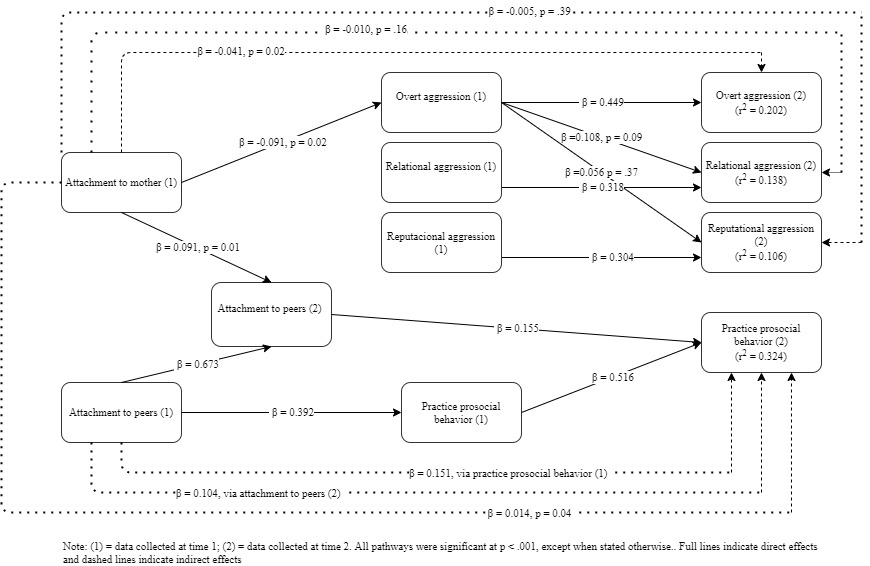


Supplementary Figure C: Generated model applied to girls


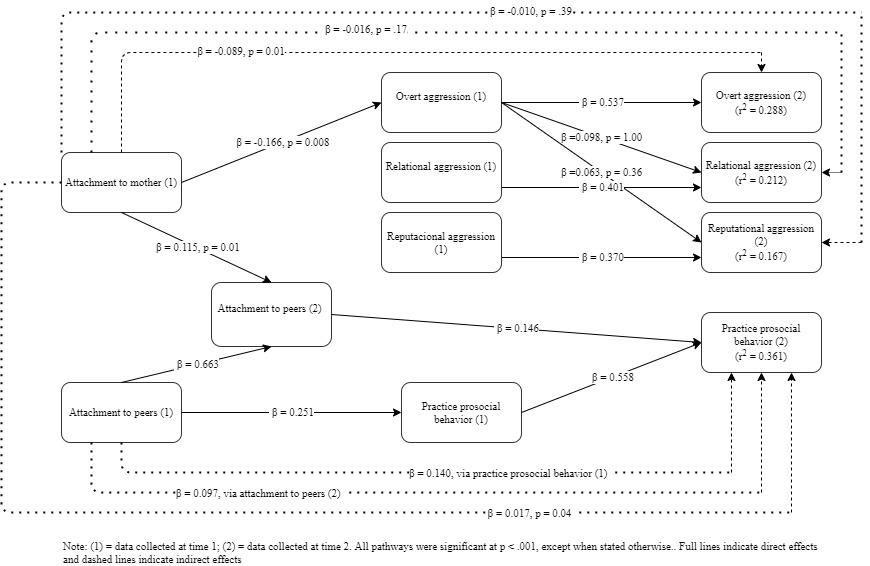


No evidence was found for invariance at the mean level, with boys and girls presenting with significant mean level differences for all measures considered in the generated model, except attachment to mother at time 1 and practice of prosocial behavior at time 1 (Supplementary Table B).

| Supplementary Table B: Descriptive values and mean comparison by gender for times 1 and 2 | | | | | | | | | | | |
| --- | --- | --- | --- | --- | --- | --- | --- | --- | --- | --- | --- |
|  | | Time 1 | | | | | Time 2 | | | | |
|  | | Boys | | Girls | |  | Boys | | Girls | |  |
|  | | M | SD | M | SD | z | M | SD | M | SD | z |
| Inventory of Parent and Peer Attachment | |  |  |  |  |  |  |  |  |  |  |
|  | Mother | 3.88 | 0.59 | 3.94 | 0.76 | -0.22 | - | - | - | - | - |
|  | Peers | 3.70 | 0.61 | 3.95 | 0.61 | -4.14^**^ | 3.67 | 0.67 | 3.92 | 0.67 | -3.61^**^ |
| Peer Experience Questionnaire Revised– bully version | |  |  |  |  |  |  |  |  |  |  |
|  | Overt aggression | 1.61 | 0.69 | 1.19 | 0.41 | -7.38^**^ | 1.63 | 0.67 | 1.17 | 0.67 | -8.33^**^ |
|  | Relational aggression | 1.57 | 0.62 | 1.34 | 0.49 | -4.16^**^ | 1.56 | 0.59 | 1.31 | 0.46 | -4.24^**^ |
|  | Reputational aggression | 1.30 | 0.60 | 1.17 | 0.40 | -2.49^*^ | 1.37 | 0.61 | 1.12 | 0.37 | -5.17^**^ |
|  | Practice of prosocial behavior | 3.01 | 0.79 | 3.18 | 0.91 | -1.79 | 2.97 | 0.73 | 3.18 | 0.83 | -2.13^*^ |
| ^**^ p ≤ .001; ^*^ p < .05 | |  | | | | |  | | | | |
